# Nicotine Keeps Leaf-Loving Herbivores at Bay

**DOI:** 10.1371/journal.pbio.0020250

**Published:** 2004-08-17

Sooner or later, a gardener looking for “nontoxic” ways to control the inevitable attack on a favorite plant will discover the nicotine remedy. Steep a cup of loose tobacco in a gallon of water, let it sit overnight, strain, and spray away. Caterpillars, aphids, and a diverse array of insects predisposed to devouring plants will soon abandon your vegetables and flowers in search of less disagreeable forage.

The ultimate sitting duck, plants rely on an arsenal of chemical metabolites to fend off predators. Many of these chemicals harbor anti-herbivore properties, which have been exploited for commercial use. Nicotine, it turns out, is so toxic that it was one of the first chemicals used in agricultural insecticides. It's not clear, though, whether these toxic metabolites are really defending plants against hungry herbivores in their natural environment, especially since many insects can tolerate various plant chemicals and sometimes even incorporate them into their own defenses. Though scientists have cataloged a long list of these presumed resistance traits, there's no evidence that they offer plants a competitive advantage against their leaf-covetous foes in nature.[Fig pbio-0020250-g001]

**Figure pbio-0020250-g001:**
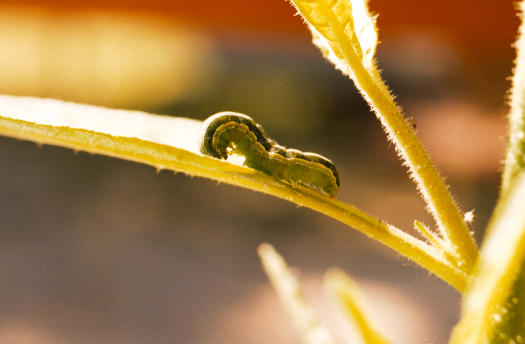
Spodoptera exigua larva feeding on Nicotiana attenuata

With plant and plant-eater engaged in an ever-escalating battle of evolutionary one-upmanship and with plants capable of producing an array of defensive responses, teasing out the predator-resistant effects of individual plant metabolites has proved challenging. Theoretically, one could track down a resistance gene by breeding plants that are genetically identical save for the gene that controls expression of a particular resistance trait. In practice, however, traditional breeding techniques aren't that precise and tend to generate additional variations in genomic regions that are linked to the target gene and that might affect resistance as well.

The tools of genetic engineering have largely overcome such limitations, allowing scientists far greater control and specificity. Following this approach, Ian Baldwin and colleagues use transgenic silencing (which introduces gene “constructs” into an organism to inactivate a gene of interest) to investigate a single resistance trait, nicotine production. Even though nicotine is one of the best-studied putative resistance traits, its specific role has been unclear.

To isolate the resistance effects of nicotine from possible confounding factors, Baldwin and colleagues blocked nicotine production in the Nicotiana attenuata tobacco plant. Focusing on an enzyme, called putrescine methyl transferase (PMT), central to nicotine biosynthesis, the authors used two techniques that interfere with PMT production by silencing the gene, *pmt*, that encodes the enzyme. One of the techniques (which adds genetic sequences called “inverted repeats” to gene fragments) proved far more effective at silencing *pmt*, producing 29 out of 34 plant lines with only 3%–4% of normal nicotine levels.

With suitably nicotine-deprived plants, Baldwin and colleagues could directly test nicotine's role in tobacco fitness. They transplanted the transgenic plants, along with nonmutant cultivated plants, in southwestern Utah, N. attenuata's native habitat. A subset of the plants was also treated with a chemical known to increase both nicotine content and resistance to herbivore attack. Predictably, several of the plant's natural insect enemies made an appearance. Untreated nicotine-deficient transgenic plants fared the worst, losing twice as much foliage to herbivores as nonmutant plants. Transgenic plants treated with the chemical boost performed much better, showing about the same amount of damage as the nonmutants. Interestingly, tobacco hornworms—which, as their name implies, feed primarily on tobacco—preferred nicotine-free plants when given the choice. Though the worms have evolved strategies for coping with nicotine's deleterious effects, these adaptations come at a price: worms feeding on nicotine-deficient tobacco grew bigger and faster than those feeding on plants with normal nicotine levels.

These results clearly show that nicotine protects tobacco plants in their native habitat, the authors conclude, and that tobacco-chewing insects “prefer low nicotine diets.” Removing nicotine from the equation reveals the relentless pressure that plants face from herbivores. Without such defenses, plants would be unceremoniously eliminated posthaste, leaving a world without greenery, not to mention oxygen. But these experiments also demonstrate the unprecedented power of transgenic tools to peel back the obfuscating layers inherent in ecological interactions to reveal the fundamental properties of those interactions. And that scientists working to unravel the tangled web of ecological interactions would do well to take advantage of the longest running experiment on earth—the natural environment.

